# *Drosophila* TDP-43 dysfunction in glia and muscle cells cause cytological and behavioural phenotypes that characterize ALS and FTLD

**DOI:** 10.1093/hmg/ddt243

**Published:** 2013-05-31

**Authors:** Danielle C. Diaper, Yoshitsugu Adachi, Luke Lazarou, Max Greenstein, Fabio A. Simoes, Angelique Di Domenico, Daniel A. Solomon, Simon Lowe, Rawan Alsubaie, Daryl Cheng, Stephen Buckley, Dickon M. Humphrey, Christopher E. Shaw, Frank Hirth

**Affiliations:** 1 Department of Neuroscience and; 2 Department of Clinical Neuroscience, Institute of Psychiatry, MRC Centre for Neurodegeneration Research, King's College London, London SE5 8AF, UK

## Abstract

Amyotrophic lateral sclerosis (ALS) and frontotemporal lobar degeneration (FTLD) are neurodegenerative disorders that are characterized by cytoplasmic aggregates and nuclear clearance of TAR DNA-binding protein 43 (TDP-43). Studies in *Drosophila*, zebrafish and mouse demonstrate that the neuronal dysfunction of TDP-43 is causally related to disease formation. However, TDP-43 aggregates are also observed in glia and muscle cells, which are equally affected in ALS and FTLD; yet, it is unclear whether glia- or muscle-specific dysfunction of TDP-43 contributes to pathogenesis. Here, we show that similar to its human homologue, *Drosophila* TDP-43, Tar DNA-binding protein homologue (TBPH), is expressed in glia and muscle cells. Muscle-specific knockdown of *TBPH* causes age-related motor abnormalities, whereas muscle-specific gain of function leads to sarcoplasmic aggregates and nuclear TBPH depletion, which is accompanied by behavioural deficits and premature lethality. TBPH dysfunction in glia cells causes age-related motor deficits and premature lethality. In addition, both loss and gain of *Drosophila* TDP-43 alter mRNA expression levels of the glutamate transporters Excitatory amino acid transporter 1 (EAAT1) and EAAT2. Taken together, our results demonstrate that both loss and gain of TDP-43 function in muscle and glial cells can lead to cytological and behavioural phenotypes in *Drosophila* that also characterize ALS and FTLD and identify the glutamate transporters EAAT1/2 as potential direct targets of TDP-43 function. These findings suggest that together with neuronal pathology, glial- and muscle-specific TDP-43 dysfunction may directly contribute to the aetiology and progression of TDP-43-related ALS and FTLD.

## INTRODUCTION

Amyotrophic lateral sclerosis (ALS) and frontotemporal lobar degeneration (FTLD) are two devastating neurodegenerative diseases for which no treatment currently exists. FTLD is a common denominator for clinical subtypes including a behavioural and two language variants, with the behavioural form being most frequent and characterized by changes in behaviour and personality, including motor abnormalities and apathy (1). ALS is characterized by the degeneration of upper and lower motor neurons, along with astrogliosis leading to muscle wasting, spasticity, paralysis of the limb and swallowing muscles, ultimately causing death of the patient within 2–5 years after diagnosis (2,3). Although FTLD and ALS can represent as rather distinct clinical entities, the discovery of aggregations of dysfunctional protein together with gene mutations identify them as a clinical continuum which may underlie common pathogenic pathways (1). Most prominent among them are TAR DNA-binding protein 43 (TDP-43) aggregates and the recently identified G4C2 hexanucleotide repeat expansion in *C9ORF72*, which account for the majority of familial ALS and FTLD cases (4–8).

TDP-43 has been identified as the major disease protein aggregating in cytoplasmic inclusions that characterize both ALS and FTLD, and mutations in the encoding *TARDBP* gene have been found in familial cases (7,8), strongly suggesting that both cytoplasmic inclusions and loss of function of TDP-43 are causally related to disease formation (9–11). TDP-43 is an evolutionarily conserved RNA-binding protein whose C-terminal part resembles a prion-like domain (12,13), which is predominantly mutated in TDP-43-related ALS and FTLD (10,11). TDP-43 is primarily expressed in the nucleus of neurons, glia cells and muscle cells, and it has been shown to regulate transcription, RNA biogenesis, splicing and RNA turnover (11,14).

Studies addressing the pathophysiological role of TDP-43 have identified a large number of RNA targets, suggesting that TDP-43 toxicity and de-regulated RNA are either directly or indirectly related to disease formation (15–18). More recent data gained in *Drosophila* and mouse revealed that both loss (i.e. nuclear clearance) and toxic gain (i.e. cytoplasmic accumulation) of TDP-43 function can contribute to disease onset and progression, even in the absence of aggregate formation (19,20). These data identified presynaptic defects as an initiating event leading to motor abnormalities and progressive neurodegeneration (19).

In contrast to growing knowledge on the neuronal function and dysfunction of TDP-43, less is known about its role in other tissues and cell types. Significantly, TDP-43 expression has also been reported in glia and muscle cells (21,22), which are equally affected in disease (2,23,24). Moreover, and in addition to neuronal inclusions, glia-specific TDP-43 aggregates have been detected in ALS and FTLD pathology (8,25–27), and sarcoplasmic aggregates of TDP-43 have been found in ALS and FTLD cases that overlap with myopathies (26,28,29). These data suggest that similar to its neuronal dysfunction, both loss and toxic gain of TDP-43 function in muscle and glial cells might be causally related to disease formation.

To address this question in a systematic way *in vivo*, we carried out high-resolution expression analysis of *Drosophila* TDP-43, TBPH, and show that similar to its human homologue, TBPH is expressed in nuclei of glia and muscle cells. We then developed a *Drosophila* model of glia- and muscle-specific TDP-43 dysfunction where we inactivated (loss of function and RNAi-specific knockdown) or overexpressed (gain of function) TBPH in either glia or muscle cells. Our results show that the gain of TBPH function in either glia or muscle cells can result in premature lethality, impaired muscle formation as well as age-related behavioural deficits that characterize ALS and FTLD. Moreover, we show that both loss and gain of *Drosophila* TDP-43 alter mRNA levels of the *Drosophila* homologues of glutamate transporters Excitatory amino acid transporter 1 (EAAT1) and EAAT2, thus linking glia and neuron pathology in TDP-43-related ALS and FTLD. Our results provide *in vivo* evidence that in addition to neuronal pathology, TDP-43 dysfunction in glia and muscle cells is causally related and hence can directly contribute to ALS- and FTLD-like pathogenesis.

## RESULTS

### *Drosophila* TDP-43, TBPH, is expressed in the nucleus of glia and muscle cells

To gain insights into the function of *Drosophila* TDP-43 in glia and muscle cells, we first determined its spatiotemporal expression pattern in these cell types. We previously reported the generation of an anti-TBPH antibody (19) that specifically detects the TBPH protein (Supplementary Material, Fig. S1). To further characterize TBPH expression in glia and muscle cells, we carried out anti-TBPH immunolabelling in the developing and adult nervous and muscular system involved in the coordination and control of motor behaviour. To detect TBPH expression in glia, we carried out co-immunolabelling with antibodies against reversed polarity (Repo) which is specifically expressed in glial cells (30–32) or in combination with glia-specific Gal4 strains, including *repo-Gal4* (33) and *MZ97-Gal4* (see Materials and methods), crossed to *UAS-Stinger-GFP* or *UAS-nLacZ, UAS-mCD8::GFP* responder lines. To detect TBPH expression in muscle, we carried out co-immunolabelling with fluorescent-conjugated Phalloidin, which specifically binds F-actin and hence visualizes muscle fibres.

**Figure 1.**
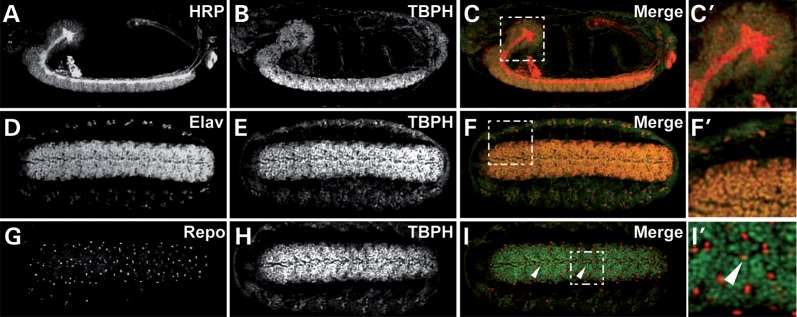
In the embryonic CNS, *Drosophila* TDP-43, TBPH, is expressed in the nuclei of neurons and glial cells. (**A**–**C**′) Confocal images of whole-mount embryos co-immunolabelled for HRP (A, white; C, red) and anti-TBPH (B, white; C, green); (C′) shows the enlarged area depicted with a dashed rectangle in (C). (**D**–**F**′) Confocal images of whole-mount embryos co-immunolabelled for the neuron-specific markers anti-ELAV (D, white; F, red) and anti-TBPH (E, white; F, green); (F′) shows the enlarged area depicted with a dashed rectangle in (F). (**G**–**I**′) Confocal images of whole-mount embryos co-immunolabelled for the glia-specific markers anti-Repo (G, white; I, red) and anti-TBPH (H, white; I, green). TBPH is expressed in the nucleus of glia cells (I, arrowheads); (I′) shows the enlarged area depicted with a dashed rectangle in (I).

**Figure 2.**
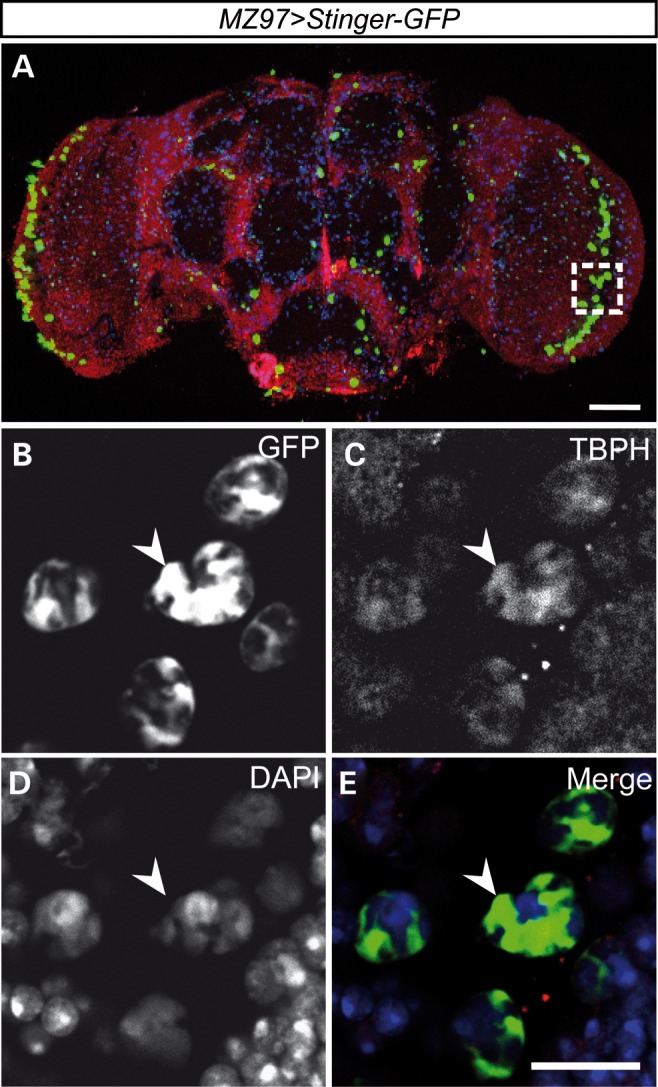
*Drosophila* TDP-43, TBPH, is seen in nuclei and peri-nuclear regions of glial cells in the adult brain. (**A**–**E**) Confocal images of whole-mount adult *MZ97-Gal4, UAS-Stinger-GFP* brain which identifies a subset of glia, including the large lamina glia. Co-labelling for GFP (green; white in B), anti-TBPH (red; white in C) and DAPI (blue; white in D). TBPH is expressed in the nucleus and the peri-nuclear region of glia cells (C–E, arrowheads). The dashed square in (A) represents the enlarged region shown in (B)–(E). Scale bar: 10 μm.

We detected TBPH immunolabelling in glial cells of the embryo (Fig. 1), larval (Supplementary Material, Fig. S2) and adult central nervous system (Fig. 2). When co-labelled with fluorescent active 4′,6-diamidino-2-phenylindole (DAPI) which binds A-T enriched nucleotide regions and thus visualizes nuclear DNA, TBPH immunoreactivity co-localized with DAPI but was also detectable outside DAPI labelling (Supplementary Material, Fig. S2, arrowheads), suggesting glia-specific TBPH expression in both nucleus and perinuclear regions. This was further supported by high-resolution confocal microscopy analysis of anti-TBPH staining of brains of *MZ97>Stinger-GFP* flies which expressed green fluorescent protein (GFP) in a subset of glial cells, including lamina glia (Fig. 2). Analysis of single confocal sections revealed TBPH labelling that co-localized with both perinuclear and nuclear Stinger-GFP as well as nuclear DAPI (Fig. 2A–E), further demonstrating that *Drosophila* TDP-43 expression in glial cells can be detected in both nucleus and cytoplasm.

**Figure 3.**
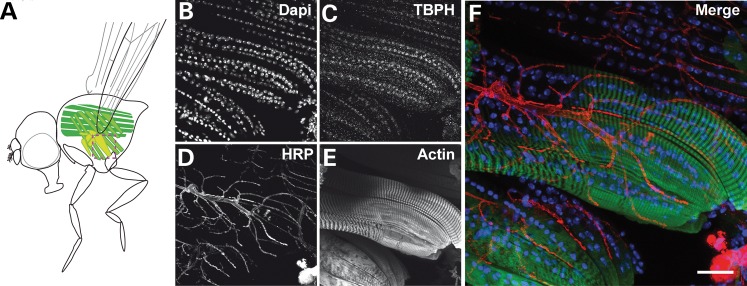
*Drosophila* TDP-43 is expressed in the nucleus of muscle cells. (**A**) Cartoon illustrating the adult flight musculature. (**B**–**F**) Confocal images of adult flight musculature co-immunolabelled for DAPI (B, white), anti-TBPH (C, white), HRP (D, white) and phalloidin (E, white). (B)–(F), enlargement of the dashed box in (A). Scale bar: 20 µm.

Next, we determined TBPH immunolabelling in larval body wall muscle and adult flight muscles. Co-labelling with phalloidin and DAPI revealed TBPH immunolabelling in all muscle cells, which exclusively co-localized with DAPI (Fig. 3), suggesting that muscle-specific TBPH expression is confined to the nucleus. These data demonstrate that similar to its human TDP-43 homologue, *Drosophila* TBPH is expressed in glia and muscle cells throughout development and adulthood; muscle expression appears restricted to the nucleus, whereas glia-specific expression can be seen in both nucleus and cytoplasm, suggesting a functional role of *Drosophila* TDP-43 in glia and muscle cells.

### Loss of *Drosophila* TDP-43 in muscle cells causes age-related motor abnormalities

To gain insights into the functional role of TDP-43 in muscle cells, we first determined whether loss of TDP-43 function causes any phenotypic alterations in muscle tissue. We therefore examined phalloidin-labelled muscle tissue in a TBPH loss-of-function background using our previously characterized mutants, *TBPH^DD96^* and *TBPH^DD100^*, which are protein null alleles (19). Careful examination of the larval musculature, however, did not reveal any gross alterations when compared with age-matched controls (Supplementary Material, Fig. S3). Also in newly hatched, 1–3-day-old adult mutant flies, we did not detect any alterations in muscle tissue (data not shown). We then wondered whether loss of TBPH may cause age-related muscle phenotypes. However, the examination of aged *TBPH*^−/−^ flies is impossible, since TBPH null mutant flies die within the first 10 days of their life due to defective steroid signalling (34). To circumvent early lethality and to gain insights into age-related phenotypes, we used the Gal4/UAS system for RNA interference-mediated targeted knockdown of TBPH. We made use of our previously established *UAS-TBPH-IR* lines that can effectively knock down TBPH (19) and crossed it with the muscle-specific *Mef2-Gal4* driver line (35) together with *UAS-Dcr2* to enhance RNAi efficacy (36).

**Figure 4.**
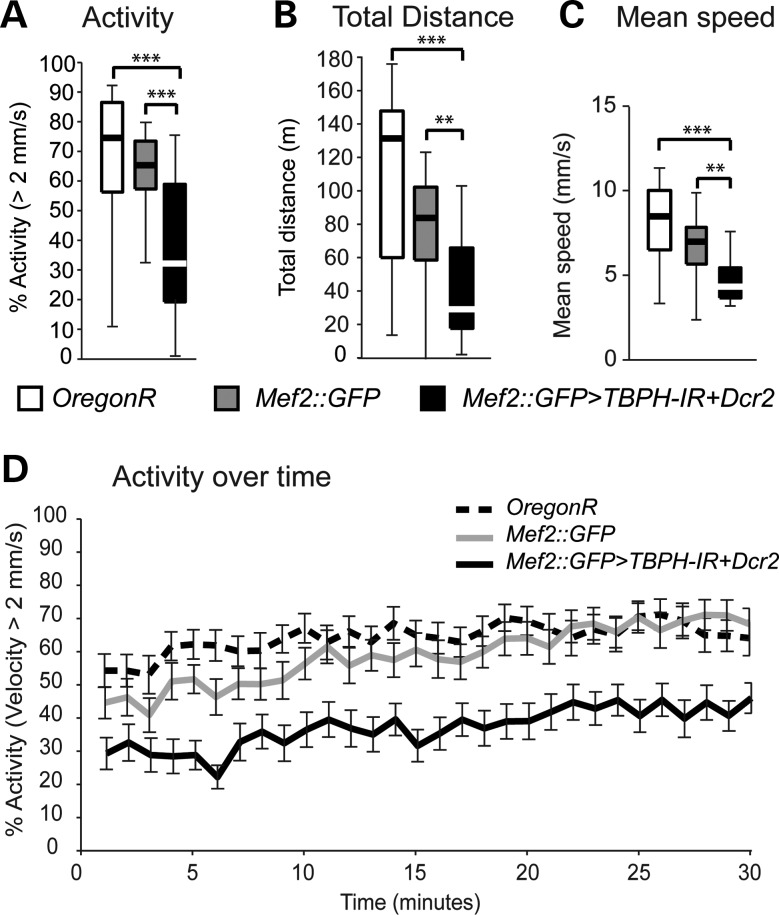
Muscle-specific knockdown of TBPH causes age-related motor abnormalities. (**A**–**D**) Open-field motion tracking of *Drosophila* with RNAi-mediated knockdown targeted to muscle cells (*Mef2::GFP>TBPH-IR, Dcr2*) affects walking activity (A), total walking distance (B), reduced mean speed (C) and activity over time (D). 30 min of tracking data for control and experimental conditions were compared using the Mann–Whitney *U*-test (*P < 0.05). Box and whisker plots (A–C) show the median (thick lines), interquartile range (boxes) and 1.5× the interquartile range (whiskers) (*n* = 24; **P < 0.01, ***P < 0.001; D, error bars, SEM).

Analysis of *Mef2::GFP>Dcr2, TBPH-IR* progeny did not reveal any differences in life cycle or survival since equal numbers of progeny derived from 150 selected embryos, when compared with *w^1118^* controls (Supplementary Material, Fig. S4A). We then carried out behavioural analysis and used startle-induced negative geotaxis as a quantifiable measure of innate escape and gravitaxis behaviours (37,38). However, analysis of *Mef2::GFP>Dcr2, TBPH-IR* and wild-type *Oregon R* control flies did not reveal any significant differences between cases and controls (Supplementary Material, Fig. S4B). We reasoned that startle-induced negative geotaxis assay might be unsuitable to detect alterations in voluntary behaviour. To address this, we used an open-field paradigm together with video-assisted motion tracking that allows numerical assessment of motor behaviours of flies (38,39). When compared with age-matched wild-type *Oregon R* and *Mef2::GFP/+* controls (Fig. 4), analysis of 30-day-old *Mef2::GFP>Dcr2, TBPH-IR* flies revealed significant alterations in overall motor activity (Fig. 4A), mean speed (Fig. 4B) and total distance travelled (Fig. 4C), and activity over time was significantly reduced as well (Fig. 4D). These data suggest that muscle-specific knockdown of *Drosophila* TDP-43 causes age-related motor abnormalities.

### Muscle-specific gain of *Drosophila* TDP-43 leads to sarcoplasmic aggregates, impaired motor behaviour and premature lethality

Cytoplasmic accumulation of TDP-43 has been observed in ALS (29) and Inclusion Body Myopathy, Paget disease and Frontotemporal Dementia (IBMPFD), which can present as a spectrum of ALS, FTLD and myopathies (40). We therefore wondered whether sarcoplasmic accumulation of normally nuclear TDP-43 may directly contribute to disease formation. To address this question, we utilized the *Mef2-Gal4* driver for muscle-specific overexpression of TBPH, using our previously characterized *UAS-TBPH* lines that can lead to reliable accumulation of TBPH in both nucleus and cytoplasm (19).

**Figure 5.**
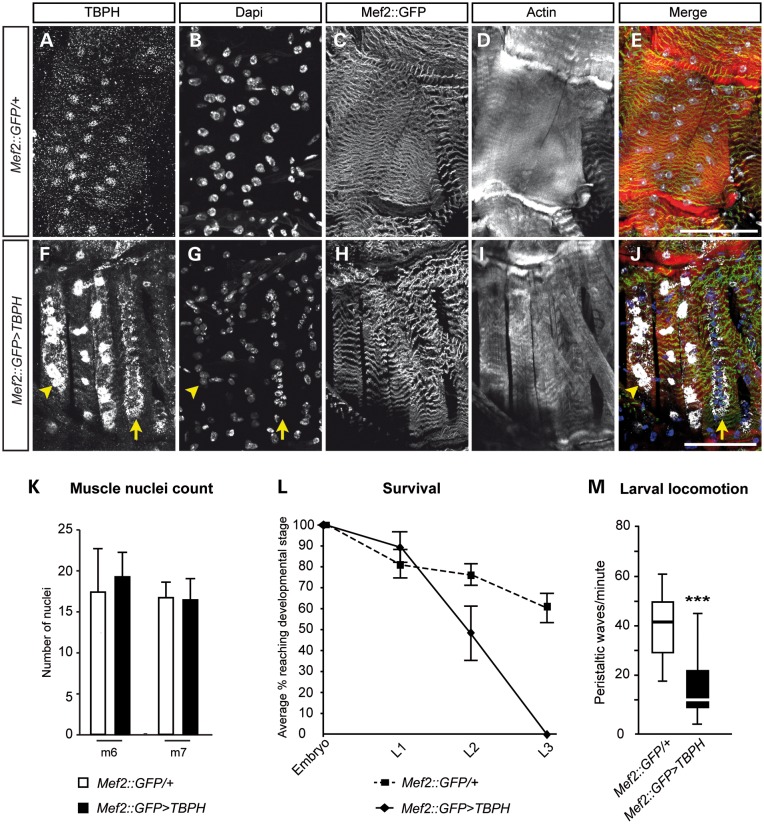
Muscle-specific gain of TBPH function leads to sarcoplasmic aggregates, impaired motor behaviour and premature lethality. (**A**–**E**) Body-wall musculature preparation of L2 larva showing muscle segment 6/7 of heterozygous *Mef2::GFP-Gal4*/+ control co-immunolabelled for anti-TBPH (A, white), DAPI (B, white), *Mef2::GFP* (C, white) and phalloidin (D, white). (**F**–**J**) Musculature of *Mef2::GFP>TBPH* gain-of-function body-wall musculature preparation of L2 larva showing muscle segment 6/7 co-immunolabelled for anti-TBPH (F, white), DAPI (G, white), *Mef2::GFP* (H, white) and phalloidin (I, white). Note that sarcoplasmic aggregates accompanied with nuclear TBPH depletion (F and J, arrow). (**K**) Nuclei counts of muscle cells in *Mef2::GFP>TBPH* gain of function for muscle 6 (m6) and muscle 7 (m7) reveal no significant changes in numbers when compared with *Mef2::GFP/+* controls (each *n* = 3). (**L**) Survival curve of embryonic and larval *Mef2::GFP>TBPH* gain of function revealing premature lethality by L3 stage. (**M**) *Mef2::GFP>TBPH* gain-of-function L2 larvae show impaired peristaltic movements. The Box and whisker plot shows the median (thick lines), interquartile range (boxes) and 1.5× the interquartile range (whiskers) (*n* = 24; ***P < 0.001; K and L, error bars, SEM). Scale bar in (E) and (J): 50 µm.

Phalloidin, anti-TBPH and DAPI triple immunolabelling of *Mef2::GFP-Gal4*-mediated *TBPH* gain-of-function mutants confirmed a significant overexpression of TBPH in muscle cells (Fig. 5F, G and J; arrowheads), when compared with age-matched controls (Fig. 5A, B and E). Intense TBPH immunoreactivity was seen in nuclei of muscle cells, and also in the sarcoplasm, albeit not in every muscle fibre. Closer inspection of immunolabelling identified sarcoplasmic TBPH aggregates surrounding DAPI-positive nuclei that were devoid of anti-TBPH immunolabelling (Fig. 5F, G and J; arrow), suggesting that gain of TBPH function led to the nuclear depletion of *Drosophila* TDP-43 in some muscle fibres. In addition, when compared with controls, DAPI-positive nuclei of *Mef2::GFP>TBPH* larval muscle fibres appeared reduced in size and alterations in phalloidin labelling could be observed (Fig. 5H and J), while the number of nuclei appeared unaltered (Fig. 5K), indicating irregular sarcomere formation during muscle development.

The observed phenotypic alterations of *Mef2::GFP>TBPH* larvae were accompanied by severe motor abnormalities and premature lethality. Thus, analysis of *Mef2::GFP-Gal4*-mediated *TBPH* gain-of-function mutants revealed that muscle-specific overexpression of *Drosophila* TDP-43 affected development and lifespan: none of the progeny eclosed as adult; the majority of mutant cases died during larval stage L2 and developed no later than L3, causing pre-pupal lethality (Fig. 5L). Examination of active *Mef2::GFP>TBPH* L2 larvae showed significantly impaired peristalsis and defective locomotion, when compared with age-matched *Mef2::GFP-Gal4/+* controls (Fig. 5M). Together, these data suggest that *Drosophila* TDP-43 dysfunction in muscles can lead to sarcoplasmic aggregates, impaired motor behaviour and premature lethality.

### TDP-43 dysfunction in glial cells causes premature lethality and age-related motor abnormalities

**Figure 6.**
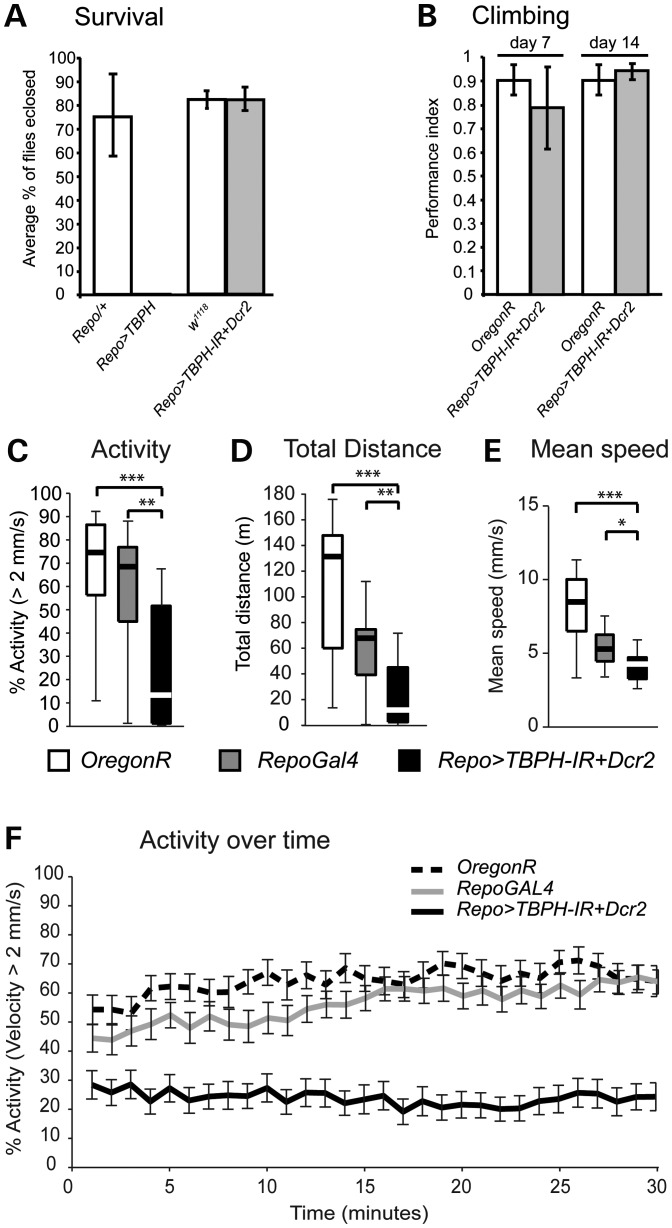
*Drosophila* TDP-43 dysfunction in glial cells leads to age-related motor abnormalities and premature lethality. (**A**) Survival analysis of glial-specific TBPH gain of function (*Repo>TBPH*) causes larval/pre-pupal lethality, whereas RNAi-mediated knockdown of TBPH (*Repo>TBPH-IR, Dcr2*) has no effect. (**B**) Startle-induced negative geotaxis analysis of day 7 and day 14 old *Oregon R* and *Repo>TBPH-IR, Dcr2* flies reveals no differences to wild-type *Oregon R* controls. (**C**–**F**) Open-field motion tracking of *Drosophila* with RNAi-mediated knockdown targeted to glia cells (*Repo>TBPH-IR, Dcr2*) affects walking activity (C), total walking distance (D), reduced mean speed (E) and activity over time (F). 30 min of tracking data for control and experimental conditions were compared using the Mann–Whitney *U*-test (*P < 0.05). Box and whisker plots (C–E) show the median (thick lines), interquartile range (boxes) and 1.5× the interquartile range (whiskers) (*n* = 24; *P < 0.05, **P < 0.01, ***P < 0.001; A and B, error bars, SD; F, error bars, SEM).

To gain insights into the functional role of TDP-43 in glial cells, we first determined whether we could observe any glia alterations in homozygous *TBPH*^−/−^ loss-of-function mutants. Examination of glia-specific anti-Repo immunolabelling of larval and adult CNS of *TBPH^DD96^*^−/−^ and *TBPH^DD100^*^−/−^ mutants did not reveal any alterations when compared with age-matched controls (data not shown). In contrast, *repo-Gal4*-mediated *TBPH* gain-of-function mutants never reached adulthood but instead died during larval stages before puparium formation, causing larval/pre-pupal arrest and death (Fig. 6A). These data demonstrate that glia-specific overexpression of TBPH function causes premature lethality.

To examine potential age-related glia phenotypes, we carried out glial-specific RNAi knockdown. *repo>Dcr2, TBPH-IR* flies showed normal development and hatched as adults without any obvious phenotypes (Fig. 6A). Moreover, analysis of *repo>Dcr2, TBPH-IR* and wild-type *Oregon R* control flies did not reveal any significant differences in innate escape and gravitaxis behaviours between cases and controls (Fig. 6B). We then tested voluntary movements of aged cases and controls in our open-field paradigm. When compared with age-matched wild-type *Oregon R* and *repo-Gal4/+* controls (Fig. 6C–F), analysis of 30-day-old *repo>Dcr2, TBPH-IR* flies revealed significant changes in overall motor activity (Fig. 6C), total distance travelled (Fig. 6D) and mean speed (Fig. 6E), and activity over time was significantly reduced as well (Fig. 6F). These data suggest that the glia-specific dysfunction of *Drosophila* TDP-43 differentially affects survival and adult motor behaviour.

### TDP-43 dysfunction alters mRNA levels of EAAT1 and EAAT2

Previous studies identified a role for astrocytes and impairment of the EAAT2, in ALS (41,42). EAAT2 is a glutamate transporter found in glia but also neurons required for glutamate clearance in the synaptic cleft, thereby protecting neurons from glutamate excitotoxicity and subsequent neurodegeneration (43). Recent genome-wide screens identified mRNA of EAAT2 and of its related orthologue EAAT1 as potential TDP-43 targets (15,16), indicating a mechanistic link between TDP-43 dysfunction and deregulated EAAT mRNA levels in ALS formation. Hence, we wondered whether *Drosophila* TDP-43 dysfunction may affect the mRNA expression level of the fly homologues of EAAT, dEAAT1 and dEAAT2 (44).

**Figure 7.**
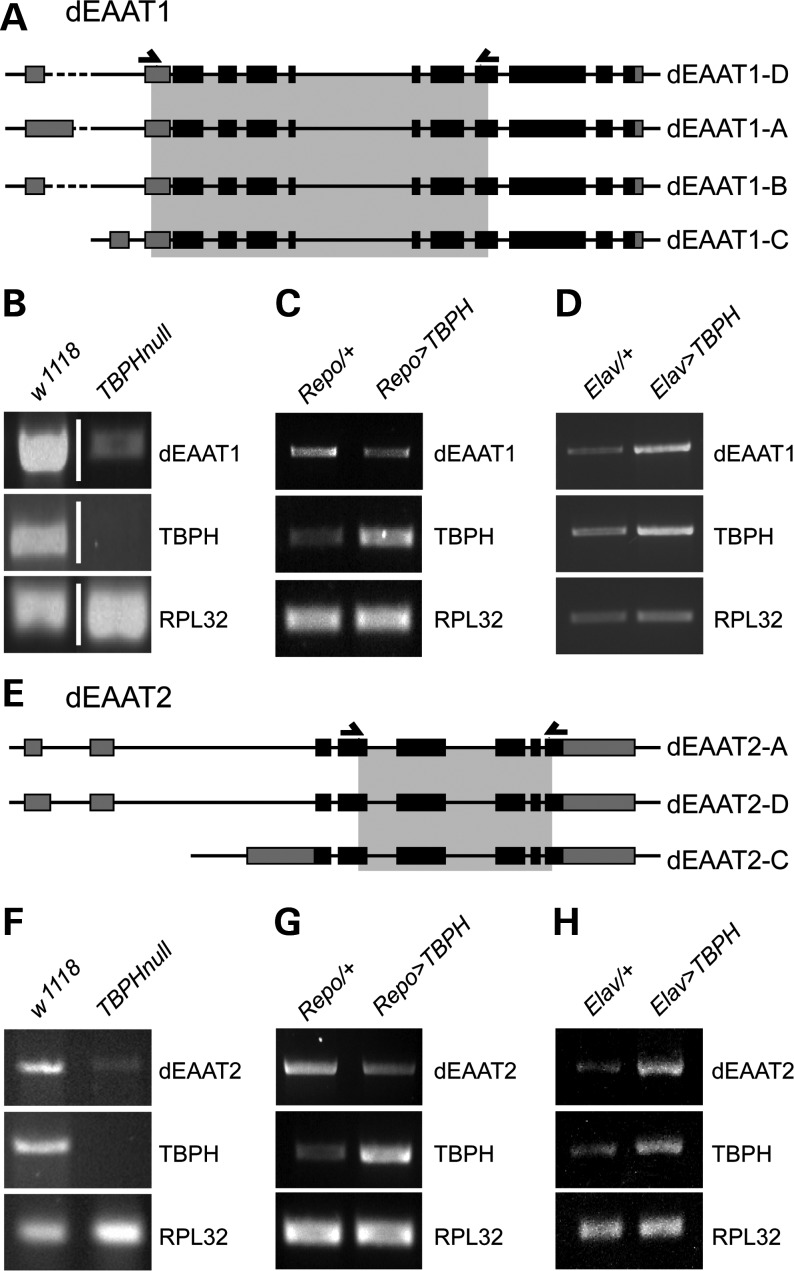
TBPH loss and gain of function leads to altered mRNA levels of dEAAT1 and dEAAT2. (**A**) Predicted isoform with exon intron structure of *Drosophila* EAAT1 (dEAAT1); arrowheads indicate used primers to amplify grey shaded fragment that covers all isoforms. (**B**) RT–PCR of dEAAT1 fragment reveals down-regulation in *TBPH* null mutant compared with *w^1118^* control (*n* = 7). (**C**) RT–PCR of dEAAT1 fragment reveals down-regulation in glial-specific *repo*>*TBPH* gain-of-function mutant compared with *repo-Gal4/+*control (*n* = 8). (**D**) RT–PCR of dEAAT1 fragment reveals up-regulation in neuron-specific *Elav*>*TBPH* gain-of-function mutant compared with *Elav-Gal4/+*control (*n* = 7). (**E**) Predicted isoform with exon intron structure of *Drosophila* EAAT2 (dEAAT2); arrowheads indicate used primers to amplify grey shaded fragment that covers all isoforms. (**F**) RT–PCR of dEAAT2 fragment reveals down-regulation in *TBPH* null mutant compared with *w^1118^* control (*n* = 4). (**G**) RT–PCR of dEAAT2 fragment reveals down-regulation in glial-specific *repo*>*TBPH* gain-of-function mutant compared with *repo-Gal4/+*control (*n* = 6). (**H**) RT–PCR of dEAAT2 fragment reveals up-regulation in neuron-specific *Elav*>*TBPH* gain-of-function mutant compared with *Elav-Gal4/+*control (*n* = 3). In all cases, controls for the TBPH expression level and the RPL32 housekeeping gene are shown.

To address this, we generated primers to amplify fragments covering all isoforms of dEAAT1 (Fig. 7A) and dEAAT2 (Fig. 7E) and carried out semi-quantitative reverse transcription polymerase chain reaction (RT-PCR) to determine their transcript levels in homozygous TBPH null mutants and TBPH gain-of-function flies. Because EAAT1 and EAAT2 are expressed in both glial cells and neurons, we overexpressed TBPH in either glial cells using *repo-Gal4* or neurons using the pan-neuronal Gal4 driver *Elav-Gal4*. Analysis of dEAAT1 transcript levels revealed a reduction in TBPH mutants in all cases examined (*n* = 7) when compared with *w^1118^* controls (Fig. 7B). In glial-specific TBPH gain-of-function flies, we observed in all cases examined (*n* = 8) a down-regulation of dEAAT1 levels when compared with heterozygous *repo-Gal4/+* controls (Fig. 7C). In contrast, neuron-specific gain of TBPH function up-regulated dEAAT1 levels in all cases examined (*n* = 7) when compared with heterozygous *Elav-Gal4/+* controls (Fig. 7D). Furthermore, analysis of dEAAT2 transcript levels revealed a down-regulation in TBPH null mutants in all cases examined (*n* = 4) when compared with *w^1118^* controls (Fig. 7F), which we also observed in all cases of glial-specific TBPH gain-of-function flies (*n* = 6) when compared with heterozygous *repo-Gal4/+* controls (Fig. 7G). In contrast, neuron-specific gain of TBPH function up-regulated dEAAT2 levels in all cases examined (*n* = 3) when compared with heterozygous *Elav-Gal4/+* controls (Fig. 7H). These data demonstrate a direct, albeit inverse, correlation between transcript levels of dEAAT1 and dEAAT2 and TBPH expression in neuron and glial cells, indicating that TDP-43 either directly or indirectly regulates glutamate transporter mRNA levels in a cell type-specific manner in the *Drosophila* nervous system.

## DISCUSSION

Our results establish that either loss or gain of TDP-43 function in muscle and glial cells can lead to cytological and behavioural phenotypes in *Drosophila* that also characterize ALS and FTLD and thus demonstrate that in addition to neuronal pathology, glial- and muscle-specific TDP-43 dysfunction can directly contribute to disease formation. These findings have implications for understanding the aetiology and progression of TDP-43-mediated ALS and FTLD.

### Muscle-specific TDP-43 dysfunction

Our findings demonstrate that similar to its human homologue, *Drosophila* TDP-43 is also expressed in the nucleus of muscle cells. Muscle-specific knockdown of *TBPH* causes age-related motor abnormalities, whereas gain of TBPH function in muscle cells can cause sarcoplasmic aggregates together with nuclear depletion of *TBPH*. These cytological phenotypes are accompanied with impaired behaviour and premature lethality. Our results thus identify a muscle-specific role of TDP-43 in *Drosophila* and show that lowered (RNAi knockdown) or increased (gain of function) levels of TBPH protein can cause pathogenic phenotypes that resemble characteristic features of ALS and FTLD.

Moreover, our data suggest that similar to its role in neuronal pathogenesis (19), equilibrated levels of TDP-43 are essential for its normal muscle-specific function. Although we observe sarcoplasmic aggregates, it remains to be shown whether aggregate formation is directly related to TDP-43 pathogenesis in muscle cells. Recent studies show that neuronal dysfunction of TDP-43 can induce cytotoxicity without aggregate formation (19,20), which may also apply to muscle-specific TDP-43 dysfunction. The observed sarcoplasmic aggregates are found in conjunction with nuclear TBPH depletion, indicating that TDP-43 may regulate its own expression by a negative feedback loop, as has been previously shown for mammalian neuronal tissue (17,45,46). Muscle-specific auto-regulation, and hence a negative feedback loop, could participate in a feed-forward mechanism whereby sarcoplasmic aggregation depletes nuclear TDP-43 function, ultimately causing a muscle-specific loss-of-function phenotype.

Previous studies showed that TDP-43 regulates a large number of RNA targets in *Drosophila,* zebrafish and mammals, which among others identified several muscle-specific targets, including dystrophin and survival motor neuron (SMN) protein (15,34,41,47). Dysfunction of dystrophin and SMN are causally related to muscle diseases in that deregulated dystrophin causes Duchenne Muscular Dystrophy (48) whereas Spinal Muscular Atrophy is caused by defective SMN function (49). Interestingly, overexpressed TDP-43 has been shown to promote exon inclusion during the splicing of SMN2 pre-mRNA (50), demonstrating a role of TDP-43 in muscle-specific RNA processing. Given the muscle-specific loss- and gain-of-function phenotypes we found in *Drosophila* and those reported in zebrafish (47), it will be interesting to determine whether deregulated TDP-43 affects RNA processing of muscle-specific target genes, thereby contributing to TDP-43-related disease formation.

### TDP-43 dysfunction in glial cells

Similar to its human homologue, we also found *Drosophila* TDP-43 expression in both nucleus and peri-nuclear regions of developing and adult glial cells. Previous studies suggested that glia pathology can contribute to ALS and neurodegeneration (23,51,52). Our results demonstrate that similar to neuronal (19) and muscle-specific (this study) dysfunction, the deregulation of *Drosophila* TDP-43 in glial cells can cause disease phenotypes. Thus, TBPH gain of function caused premature lethality, whereas RNAi-mediated knockdown caused age-related motor abnormalities.

Recent *in vitro* experiments using familial ALS patient-derived induced pluripotent stem (IPS) cells with the M337V TDP-43 mutation showed that astrocytes generated from these IPS cells reveal cytoplasmic inclusions and premature cell death, thereby mimicking histopathological findings of TDP-43 proteinopathies (53). Comparable observations have recently been made in *Drosophila* (54), suggesting that similar to our observations in muscle-specific TDP-43 dysfunction, ALS- and FTLD-like phenotypes concur with cytoplasmic aggregates in glia cells. It remains to be shown, however, whether glial-specific aggregate formation affects nuclear TDP-43 expression, hence causing a loss-of-function phenotype, or whether cytoplasmic inclusions represent a cellular protection mechanism indirectly related to TDP-43 toxicity (19,55).

In addition to the observed behavioural phenotypes, we also identified dEAAT1 and dEAAT2 as potential direct targets of *Drosophila* TDP-43. Thus, RT–PCR analysis revealed that both loss and gain of TBPH affect dEAAT1 and dEAAT2 RNA levels, in that TBPH loss of function caused a decrease, whereas its glial-specific gain of function caused a decrease in dEAAT1 and dEAAT2 transcript levels, while neuron-specific gain of function caused an increase in dEAAT1 and dEAAT2 transcript levels. These data suggest that equilibrated levels of *Drosophila* TDP-43 regulate the level, stability and/or post-transcriptional modification of glutamate transporter mRNA likely via distinct, cell type-specific mechanisms as indicated by the inverse regulation of dEAAT1 and dEAAT2 transcript levels in neuron versus glial-specific overexpression of TBPH. Our results are consistent with previous findings, showing that EAAT1 and EAAT2 levels (56), as well as EAAT2 RNA processing (57–59) are significantly altered in ALS patients. Defective EAAT function has been linked to glutamate excitotoxicity, which also characterizes ALS (42). Moreover, RNA sequencing screens identified EAAT1 and EAAT2 as targets of TDP-43 (15,16). These data together with our findings in *Drosophila* suggest that defective EAAT function may contribute to TDP-43-mediated pathogenesis in ALS and FTLD.

In summary, we have demonstrated that comparable with its neuronal dysfunction (19), glia- and muscle-specific loss and gain of TDP-43 function can cause cytological and behavioural phenotypes in *Drosophila* that characterize ALS and FTLD. Given the striking similarities in expression and function of *Drosophila* and human TDP-43 (12,19), our findings suggest that TDP-43 dysfunction in glial and muscle cells directly contribute to the aetiology and progression of TDP-43 related ALS and FTLD.

## MATERIALS AND METHODS

### Fly stocks

Fly stocks were maintained at 25°C in a 12 h light/dark cycle in a humid incubator (LMS) on standard cornmeal food, unless for aging experiments where aged flies were maintained on 15% sugar/yeast medium (38,39). The following strains were used: *Oregon R* (wild-type); *w^1118^*; *TBPH^96^*^−/−^ and *w^1118^*; *TBPH^100^*^−/−^ (19); w[*]; P{w[+mC] = GAL4-elav.L}3 [Bloomington stock BL8760]; *repo-Gal4* (33); *UAS-mCD8::GFP*; *UAS-Dcr2* (36); *UAS-TBPH* (19); *UAS-TBPH-IR* (19); *Mef2::GFP-Gal4* (35), *MZ97-Gal4,UAS-Stinger-GFP* (a gift from B. Altenheim).

### Reverse transcription–PCR

Four flies (*TBPH^96^*^−/−^), 20 adult heads (Elav>TBPH) or 5 whole L3 (Repo>TBPH) per genotype were homogenized using a pestle (Fischer) in 500 µl of Trizol (Invitrogen) until no body structures were identifiable. RNA extraction using Trizol was performed following the manufacturer's instructions. RNA was resuspended in nuclease-free (DEPC-treated) H_2_O using 1 µl of water per 10 µl of Trizol. RNA content was measured using a NanoDrop (Thermo Scientific); concentrations were typically 150–300 ng RNA/µl H_2_O. Isolated RNA was treated with DNA-free (Ambion) to remove contaminating DNA following the manufacturer's instructions.

For the RT reaction, 1 µg of DNase-treated RNA was amplified for 60 min at 37°C using mouse megalovirus reverse transcriptase (M-MLV RT; Promega) and random hexamer oligonucleotide primers (Fermentas) following the manufacturer's instructions. M-MLV RT was inactivated by heating to 70°C for 15 min. cDNA was stored at −20°C for later use.

To measure d*EAAT1* and *dEAAT2* mRNA levels, cDNAs were amplified using PCR in a series of increasing cycle numbers to obtain the linear phase of amplification. PCRs were carried out to compare *dEAAT1* and *dEAAT2* transcripts in experimental conditions. As positive and negative controls in *w^1118^*, *TBPH^96^*^−/−^ loss- or *TBPH* gain-of-function mutants, equal amounts of cDNA present in the starting reaction was confirmed by measuring *TBPH* and *RPL32*. Primers used were:

(i) TBPH: forward primer, 5′-TGGCCCAGATCAAGAAGGAC-3′; reverse primer, 5′-TTACCTCGGTGGTGTCCGTT-3′,

(ii) dEAAT1: forward primer, 5′-TCGGAATCGAGGGAGGAGATAGGC-3′; reverse primer, 5′- GGCAACACCCAGCGGGGAAA-3′,

(iii) dEAAT2: forward primer, 5′-ATAGCGCTGCGCACGTTGGTT-3′; reverse primer, 5′-CACCGAGTCCGTCATTGTCA-3′,

(iv) RPL32: forward primer, 5′-CGCCGCTTCAAGGGACAGTATC-3′; reverse primer, 5′-CGACAATCTCCTTGCGCTTCTT-3′.

Amplified DNA was separated by electrophoresis in 1.1% agarose. DNA was visualized using ethidium bromide and digitally imaged by a computer mounted camera. Grey value of the DNA bands captured in the digital gel images were measured using the plot profile in FIJI image processing package (http://pacific.mpi-cbg.de).

### Immunohistochemistry

Embryos were collected on fruit agar plates and dechorionated in 50% sodium hypochlorite. Washed embryos were fixed in Heptane/PIPES EGTA MgSO_4_ formaldehyde solution (100 mm piperazine-*N*,*N*′-bis(2-ethanesulfonic acid), 2 nm ethylene glycol tetraacetic acid, 1 nm MgSO_4_, pH 7+3.7% formaldehyde) for 30 min. Embryos were then devitellinized in methanol. For immunohistochemistry, embryos were washed in 0.1% PBS-Triton (PBT) [1.86 mm NaH_2_PO4, 8.41 mm Na_2_HPO_4_, 175 mm NaCl, 0.1% Triton X-100 (Fluka), pH 7.4] + bovine serum albumin (Sigma) before blocking in PBT-5% NGS (normal goat serum, Invitrogen). Embryos were incubated overnight at 4°C in 100 µl of PBT-NGS and the appropriate antibody dilution and washed in PBT then PBT-NGS before incubating overnight in100 µl of PBT-NGS and the appropriate secondary antibody dilution. Embryos were then washed in PBT and incubated in Vectorshield mounting medium with DAPI (Vector Laboratories) overnight before mounting on glass slides. Larval and adult CNS protocols were carried out as described previously (38). Neuromuscular junction dissections were carried out according to the established protocol (60). Primary antibodies used were: mouse anti-Repo (1:20) and rat anti-Elav (1:30) and mouse 3C11 (anti-synapsin, 1:100); all obtained from the Developmental Studies Hybridoma Bank (DSHB) under the auspices of the NICHD and maintained by The University of Iowa; rabbit anti-TBPH antibody (1:3000); goat anti-horseradish peroxidase (HRP)Cy3 (1:100; Stratech) and 488-Phalloidin (1:1000; Invitrogen). Secondary antibodies were Alexa fluor 488, 568 and 647 (each 1:150; Invitrogen).

### Image acquisition and analysis

Images were obtained with either Motic BA400 or Leica TCS SP5 confocal microscope with Leica Application Suite Advanced Fluorescence (LAS AF) version 2.0.2 software. For confocal images, channels were scanned sequentially, and confocal z-stacks were processed using FIJI. Figures were arranged using Adobe Illustrator and Adobe Photoshop.

### Embryo and larval vitality

Fifty embryos were placed on an apple juice agar plate. Hatched larvae were counted 24 h later. Results of six independent experiments are shown. For L1–L3 survival, 50 first instar larvae were put on an apple juice agar plate with yeast paste. The number of L2 larvae was counted 24 h after placing L1 larvae. L3 larvae were counted 72 h after placing L1 larvae. The mean and SD of triplicates are shown.

### Eclosion analysis

Embryos were collected on fruit agar plates in 5 h batches and left in a 25°C incubator until larvae hatched. Fifty L1 larvae were placed in a vial and a tally made of all fully eclosed adult flies, mean values were calculated and plotted as a percentage of the total number of larvae picked. Significance was calculated using an unpaired *t*-test. This was carried out in triplicate for each genotype.

### Larval motility

Thirty wandering third instar larvae (or second instar larvae for *Mef2::GFP>TBPH* and *Mef2::GFP/+*) were individually placed on a fruit agar plate and allowed to recover for 30 s. The number of peristaltic waves, travelling in either direction, was scored over 1 min. Significance was calculated using an unpaired *t*-test (two-tailed). Equal variance assumptions were based on Levene's test for equality of variance.

### Startle-induced negative geotaxis

Startle-induced negative geotaxis tests were carried out in adapted 25 ml serological pipettes, the bottom sealed with parafilm and the top cut-off and bunged with cotton wool. The ‘start line’ was the 2 ml point, and the ‘finish line’ was the 25 ml point. Forty-five female flies aged 1–3 days were tested for each genotype: 3 pipette tubes had 15 flies in each. Flies were tapped to the bottom of the tube and allowed to climb for 45 s, after which the number of flies at the bottom (below start line) and top (above finish line) were counted. This was carried out four times on each tube of flies, the first results disregarded. A performance index (PI) was calculated for each group of 15 flies (3 trials), and the mean PI for the three groups of flies for each genotype was subsequently calculated.
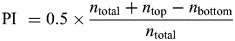

where *n*_total_ is the total number of flies, *n*_top_ the total number of flies at the top and *n*_bottom_ the total number of flies at the bottom. If all flies climb to the top of the tube, the score is 1, and if no flies climb the score is 0. Equal variance assumptions were based on Levene's test for equality of variance.

### Video-assisted motion tracking

Tracking arenas were modified 6-well tissue culture plates (35 mm diameter wells) filled with silicon rubber (Sylguard) to leave a 3 mm space so that flies could walk freely but not hop or fly. 18–24 flies were briefly anaesthetized with CO_2_, placed in separate arenas and left to recover at 25°C for 45 min before being placed above an array of white light emitting diodes within a temperature-controlled incubator. Tracking was carried out at 25°C. A black and white charge coupled device camera (Hitachi, MP-M1A) positioned above the arenas was connected to a PC via an analogue capture card (Integral Technologies, Flashbus MV Lite). Recordings were carried out during the same time slot. Recorded videos were converted to fly movie format using the motmot package (61) and loaded into Ctrax software (62) to analyse the positions of the flies throughout the video. Position data for the 30-min file was exported as a Matlab-compatable (Mathworks) matrix file. Errors in the tracking were fixed using Matlab (Mathworks) as well as FixErrors GUI (62), which is described in further detail at http://ctrax.sourceforge.net/fixerrors.html. Fixed trajectories were analysed in Matlab using custom scripts (written by D.M.H.). This analysis determined the mean velocity, mean activity, activity over time and mean cumulative distance travelled by the population of flies in the arena. Activity was defined as movement per frame above a velocity of 2 mm/s. The average activity was the percentage of frames where the fly was active (>2 mm/s velocity). The mean velocity was the average of velocities in each frame of the recording only when the fly was active. Box plots were generated in Matlab where the boxes show the median and upper and lower quartiles; whiskers contain data 1.5× the interquartile range; the plus symbol indicates a data point within 3× the interquartile range. Significance was calculated using the Mann–Whitney *U*-test with a Bonferroni correction to account for multiple comparisons.

### Statistical analysis

Statistical analysis was carried out using SPSS 15.0 for Windows. The alpha level for all tests was 0.05. For details of the statistical tests used and the actual *P*-values obtained, see Supplementary Material, Table S1.

## SUPPLEMENTARY MATERIAL

Supplementary Material is available at *HMG* online.

## FUNDING

This work was supported by the UK Medical Research Council (G-070149 to F.H.), the Royal Society (Hirth/2007/R2 to F.H.), the Motor Neurone Disease Association (Hirth/Oct07/6233 and Hirth/Mar12/6085 to F.H. and C.E.S.), Parkinson's UK (G-0714 to F.H.), Alzheimer's Research UK (ARUK-PhD2012–18 to F.H. and C.E.S.) and the Fondation Thierry Latran (2/2011/DrosALS to F.H.). Funding to pay the Open Access publication charges for this article was provided by the Medical Research Council UK and King's College London.

## Supplementary Material

Supplementary Data
