# Supplementary material for: Drosophila TDP-43 dysfunction in glia and muscle cells cause cytological and behavioural phenotypes that characterize ALS and FTLD
Source: Hum Mol Genet. 2013 May 31;22(19):3883–93. doi: 10.1093/hmg/ddt243 (PMC3766182; doi:10.1093/hmg/ddt243)
Supplement: Supplementary Data [file supp_22_19_3883__index.html]

Drosophila TDP-43 dysfunction in glia and muscle cells cause cytological and behavioral phenotypes that characterize ALS and FTLD — Drosophila TDP-43 dysfunction in glia and muscle cells cause cytological and behavioural phenotypes that characterize ALS and FTLD — Drosophila TDP-43 dysfunction in glia and muscle cells cause cytological and behavioural phenotypes that characterize ALS and FTLD — Supplementary Data 

# *Drosophila* TDP-43 dysfunction in glia and muscle cells cause cytological and behavioural phenotypes that characterize ALS and FTLD

## Supplementary Data

Supplementary Data

**Files in this Data Supplement:**

- Supplementary Data - Pdf file
