# Supplementary material for: Drosophila TDP-43 dysfunction in glia and muscle cells cause cytological and behavioural phenotypes that characterize ALS and FTLD
Source: Hum Mol Genet. 2013 May 31;22(19):3883–93. doi: 10.1093/hmg/ddt243 (PMC3766182; doi:10.1093/hmg/ddt243)
Supplement: Supplementary Data [file supp_ddt243_ddt243supp.pdf]

HMG-2013-D-00457
Revised manuscript

SUPPLEMENTARY MATERIAL

Drosophila TDP-43 dysfunction in glia and muscle cells cause cytological and behavioral phenotypes that characterize ALS and FTLD

Danielle C. Diaper[1], Yoshitsugu Adachi[1], Luke Lazarou[1], Max Greenstein[1], Fabio A. Simoes[1], Angelique Di Domenico[1], Daniel A. Solomon[1], Simon Lowe[1], Rawan Alsubaie[1], Daryl Cheng[1], Stephen Buckley[1], Dickon M. Humphrey[1], Christopher E. Shaw[2], and Frank Hirth[1,*]

[1]Department of Neuroscience, and [2]Department of Clinical Neuroscience, Institute of Psychiatry, MRC Centre for Neurodegeneration Research, King's College London, London, SE5 8AF, United Kingdom.

*Correspondence to: Dr. Frank Hirth, Department of Neuroscience, Institute of Psychiatry, King’s College London, PO Box 37, 16 De Crespigny Park, SE5 8AF, London, United Kingdom; Tel: ++44 20 7848 0786; Fax: ++44 20 7708 0017; email: Frank.Hirth@kcl.ac.uk

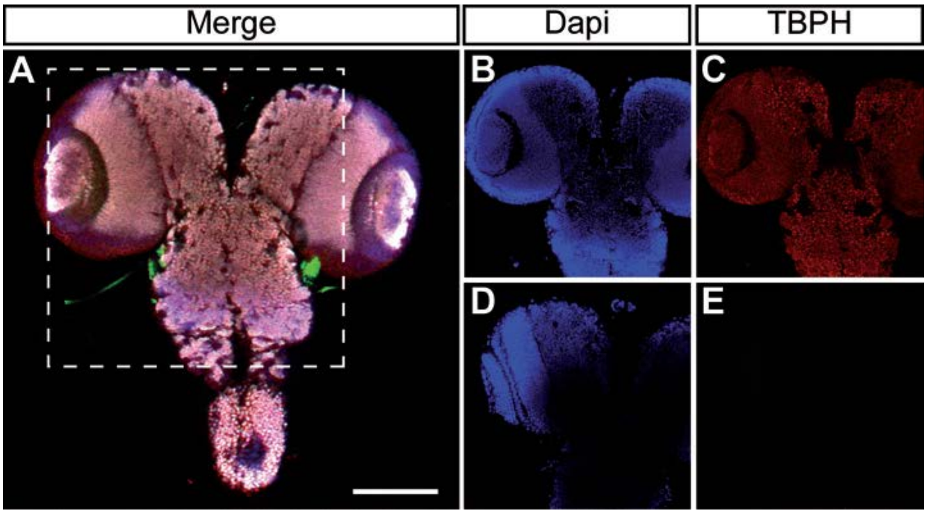

Figure S1. A polyclonal antibody against *Drosophila* TDP-43, TBPH, specifically recognizes TBPH. The specificity of the anti-TBPH antibody was confirmed via immunohistological staining of larval CNS of *TBPH-/-* null mutants and heterozygous *TBPH+/-* controls. (**A-C**) Heterozygous deletion mutants show TBPH expression throughout the larval CNS, whereas (**D, E**) TBPH null flies do not show any fluorescent signal using the anti-TBPH antibody. Images are single z-slices with 1 µm step size. Scale bar: 50 µm.

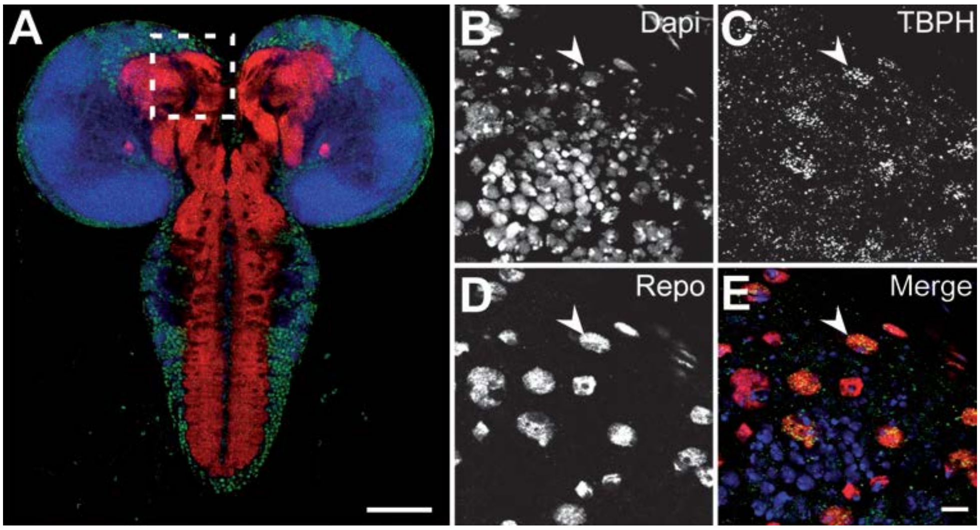

Figure S2. *Drosophila* TDP-43, TBPH, is expressed in the nucleus of glial cells during larval development. (**A-E**) Confocal image of whole-mount L3 larval CNS immunolabelled for the synaptic marker 3C11 (red in **A**), DAPI (blue; white in **B**; blue in **E**), anti-TBPH (green; white in **C**, green in **E**), and the glia-specific marker anti-Repo (white in **D**, red in **E**). Arrowheads indicate co-localisation of TBPH with Repo in the nucleus of glial cells. The dashed square in **A** represents the enlarged region shown in **B-E**. Scale bars; 10µm (B-E); 50µm (A).

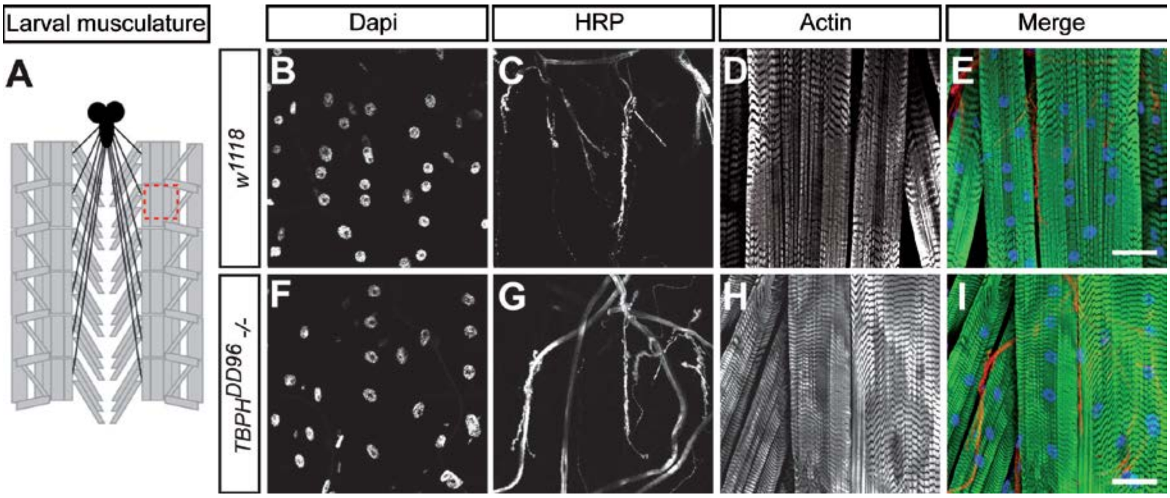

Figure S3. Loss of TBPH does not affect larval body wall musculature. **A** Body-wall musculature preparation of L3 larva. (**B-E**) *w^{1118}* control musculature co-immunolabelled for DAPI (**B**, white), HRP (**C**, white), and phalloidin (**D**, white) shown for muscle segment 6/7. (**F-G**) Musculature of homozygous *$TBPH^{DD96-/-}$* null mutant co-immunolabelled for DAPI (**F**, white), HRP (**G**, white), and phalloidin (**H**, white) shown for muscle segment 6/7. Phalloidin staining of F-actin (D, H) visualizes normal sarcomere structure in TBPH null larvae. B-E, enlargement of box in A. Scale bar; 50μm.

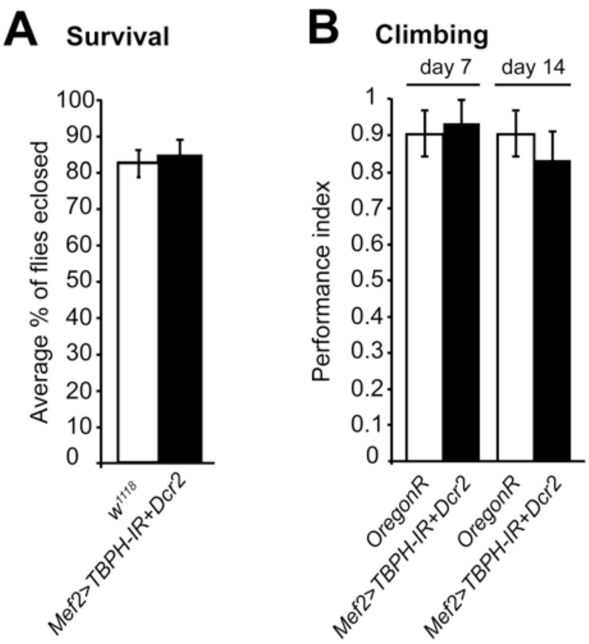

Figure S4. Muscle-specific RNAi knockdown of TBPH does not affect survival and startle-induced negative geotaxis. (**A**) Survival analysis of muscle-specific TBPH RNAi knockdown (*Mef2>TBPH-IR, Dcr2*) does not affect survival, compared to w^{1118} control. (**B**) Startle-induced negative geotaxis analysis of day 7 and day 14 old *Mef2>TBPH-IR, Dcr2* flies reveals no differences to age-matched wildtype *Oregon R* controls. (n=3; error bars, SD).

Supplementary Table 1. Statistical tests used and their results shown in respective figures (alpha value = 0.05).

Figure	Test used	Software (version)	Comparison	n	P value	Sig.	Other values
Fig 5A	Mann-Whitney U-test, Bonferroni correction	Matlab (7.10.0)	Activity: OregonR, Mef2>TBPH-RNAi+Dcr2	24,24	2.03 x 10-4	***	
Fig 5A	Mann-Whitney U-test, Bonferroni correction	Matlab (7.10.0)	Activity:`Mef2Gal4, Mef2>TBPH-RNAi+Dcr2	24,24	9.7 x 10-5	***	
Fig 5A	Mann-Whitney U-test, Bonferroni correction	Matlab (7.10.0)	Total distance: OregonR, Mef2>TBPH-RNAi+Dcr2	24,24	4.1 x 10-5	***	
Fig 5B	Mann-Whitney U-test, Bonferroni correction	Matlab (7.10.0)	Total distance: Mef2Gal4, Mef2>TBPH-RNAi+Dcr2	24,24	0.0046	**	
Fig 5C	Mann-Whitney U-test, Bonferroni correction	Matlab (7.10.0)	Mean speed: OregonR, Mef2>TBPH-RNAi+Dcr2	24,24	2.8 x 10-5	***	
Fig 5C	Mann-Whitney U-test, Bonferroni correction	Matlab (7.10.0)	Mean speed:Mef2Gal4, Mef2>TBPH-RNAi+Dcr2	24,24	0.0047	**	
Fig 6L	T-test	Graphpad Quickcalcs	Larval locomotion: Mef2Gal4/+, Mef2>TBPH	30,30	<0.0001	***	t = 8.6584, df = 58
Fig 7C	Mann-Whitney U-test, Bonferroni correction	Matlab (7.10.0)	Activity: OregonR, Repo>TBPH-RNAi+Dcr2	24,24	6.6 x 10-6	***	
Fig 7C	Mann-Whitney U-test, Bonferroni correction	Matlab (7.10.0)	Activity:RepoGal4, repo>TBPH-RNAi+Dcr2	24,24	0.0028	**	
Fig 7D	Mann-Whitney U-test, Bonferroni correction	Matlab (7.10.0)	Total distance: OregonR, repo>TBPH-RNAi+Dcr2	24,24	1 x 10-6	***	
Fig 7D	Mann-Whitney U-test, Bonferroni correction	Matlab (7.10.0)	Total distance: RepoGal4, Repo>TBPH-RNAi+Dcr2	24,24	0.0023	**	
Fig 7E	Mann-Whitney U-test, Bonferroni correction	Matlab (7.10.0)	Mean speed: OregonR, Repo>TBPH-RNAi+Dcr2	24,24	1.4 x 10-5	***	

Fig 7E	Mann-Whitney U-test, Bonferroni correction	Matlab (7.10.0)	Mean speed: RepoGal4, Repo>TBPH-RNAi+Dcr2	24,24	0.0427	*	
